# Exploring the Gantt chart as a tool to highlight double report in case series published during the first wave of the COVID-19 pandemic

**DOI:** 10.1186/s13643-022-02024-0

**Published:** 2022-07-30

**Authors:** Vânia N. Hirakata, Maria Lúcia R. Oppermann, Vanessa K. Genro, Angela J. Reichelt

**Affiliations:** 1 Unidade de Bioestatística, Hospital de Clínicas de Porto Alegre, Porto Alegre, Brazil; 2 Serviço de Ginecologia e Obstetrícia, Hospital de Clínicas de Porto Alegre and Faculdade de Medicina, Universidade Federal do Rio Grande do Sul, Porto Alegre, Brazil; 3 Serviço de Ginecologia e Obstetrícia, Hospital de Clínicas de Porto Alegre, Porto Alegre, Brazil; 4 Serviço de Endocrinologia e Metabologia, Hospital de Clínicas de Porto Alegre, Porto Alegre, 90035-903 Brazil

**Keywords:** Gantt chart, Double reporting, Case report, Case series, National registries, Research reporting

## Abstract

**Background:**

During the COVID-19 pandemic, some studies describing different aspects of the infection included very similar participants, rising suspicion about double reporting. We aimed to evaluate the Gantt chart as a tool to highlight possible double reporting. The chart is routinely used in business applications to depict tasks of a project, by plotting horizontal bars against time, showing their time span and overlaps.

**Methods:**

All case reports and case series of pregnant women with COVID-19, published by July 15, 2020, were included. Initial and final dates of participants’ enrollment, country, city, hospital, and number of pregnancies were plotted in the Gantt chart. Bars stand for enrollment dates of each study, according to hospital and city, thus allowing comparisons.

**Results:**

We included 116 articles in the present analysis. The Gantt chart highlighted papers in which some participants were likely the same, thus allowing easier identification of double reporting of cases. Combining all information and pregnancy characteristics and outcomes helped to recognize duplications when the authors did not acknowledged the previous publication.

**Conclusions:**

Unintended double reporting may occur, especially in exceptional times. The Gantt chart may help researchers to visually identify potential duplications, thus avoiding biased estimates in systematic reviews or meta-analysis.

**Supplementary Information:**

The online version contains supplementary material available at 10.1186/s13643-022-02024-0.

## Background

The novelty of the COVID-19 illness and the search for rapid answers to questions on its characteristics, treatment, and prognosis unleashed a flood of papers being rapidly produced, some with no strict adherence to methodological directives [1]. Several systematic reviews on pregnancy and COVID-19 were available by the end of 2020, and concern about case duplications and other methodological issues was raised at that time [2]. An editorial called attention to the report of different aspects of the disease in the same participant, in papers published in different journals, with no clear citation of a prior publication [3].

Double reporting is not particular to the COVID-19 pandemic. It has been described at least since the 1980s; at that time, the main concern was about publishing the same article in different printed journals, essentially, an ethical problem [4]. With the advent of data synthesis studies, including systematic reviews with meta-analysis of randomized controlled trials (RCTs), the effects of double reporting on estimates of effect size became a scientific matter [5, 6], added to the ethical issues.

That scientific problem posed a challenge: how to detect overlapping cases when performing a systematic review? Our study aimed to evaluate the Gantt chart as a tool to identify and highlight such possible duplications.

## Methods

This is a secondary analysis of data collected for a systematic review [7]. The review project was approved on 18 August 2020 by the Research Ethics Committee of the Hospital de Clínicas de Porto Alegre, Brazil (CAAE 35017020600005327), under number 2020-0382. The original systematic review was carried out in accordance with relevant guidelines and regulations [8, 9]. We have not personally included any patient in the present study, nor in the original review; all data were collected from published studies (references in Additional file 1). Thus, informed consent form was not deemed necessary.

Studies encompassed in this manuscript were published in 2020. We searched for articles up to July 3, in PubMed and Embase, and July 15, in medRxiv and the Cochrane Excel sheet “Perinatal outcomes in COVID-19 infection” [10]. During data extraction for the systematic review, we became aware of potential participant duplication in some studies. Therefore, we searched for a tool that might expose the suspected duplications through bar graphs; the Gantt chart appeared a suitable choice.

Originally intended to plot the components and time frames of several activities of a complex project, the Gantt chart is a bar graph mainly used in business administration [11]. Although in business applications it presents the interrelationships of different tasks of a project across time, here each study stood for a “different task.” The Gantt chart differs from a conventional bar chart because time can be included as a variable; this allows researchers to visualize the time span of enrollment or duration of each study. The Gantt chart data were drawn from a spreadsheet; the insertion of initial and final dates of participants’ enrollment in each study generated a bar at the right side of the chart — the duration of the study/“task.” The Gantt chart can also highlight the “hierarchy” of studies, showing which ones could have participants already included in a larger or prior study during a parallel time frame. Here, national registries can be viewed as the “parent” studies and the smaller case series as “sub-activities” [11].

The following data from each study were plotted: continent, followed by country, municipality/city, hospital, author, number of pregnancies, and dates of the participants enrollment, as provided by the authors. When just the month was provided, we set the whole month as dates; if authors declared early in the month, we set day as 5, mid-month as day 15, and late month as day 25.

Studies from the same hospital were grouped and compared to search for overlap of dates and authors. If overlap of dates occurred, clinical characteristics of individual cases (when available), or of the whole case series, were compared among studies. These comparisons included information about maternal and gestational ages, date and mode of delivery, complications of delivery or of COVID-19, laboratory results, treatments, and outcomes. Additional information on the newborn helped to further refine the data. For our systematic review, all case series from the same hospital within the same date frame were discarded, except for the largest one presenting the outcome of interest. We included all case series from the same hospital when there was no overlap of data. Whenever a national registry was included, overlap was presumed if dates reported in the smaller studies were, partially or entirely, within the time range covered by the national registry. If individual data were provided in any of the series to be discarded, the cases clearly not doubly reported in different papers were included in data synthesis.

Authors were contacted by e-mail if dates of data collection were not fully reported or if case series in the same period coincided with a national registry that could include the same participants. Studies were excluded if dates were not provided in the original paper or upon request.

For better visualization of a potential overlap, different shades of the colors were used for the horizontal bars of the chart, each representing one hospital. Each color spectrum (e.g., light green, medium green) represented a country. National registries were always the “parent” bar and were represented by the darkest color of the spectrum.

The outcome chosen in the original meta-analysis was rare — the frequency of endocrine disorders in pregnant women with COVID-19; it could be retrieved only after a thorough reading of almost all the articles. To plot the chart in the current manuscript, we did not take the outcome of interest into consideration, since our intention was to show how to organize the studies in a logical order to evaluate potential duplications. Therefore, apart from this main analysis, we performed an exercise to illustrate the use of the Gantt chart when the outcome is more frequent. We chose C-section rates in women with COVID-19 described in studies from New York City hospitals to show how numbers could be impacted.

Statistics were performed with Excel® spreadsheet and SPSS® version 18. Descriptive analyses were expressed as number (percentage).

## Results

For the present analyses, 132 articles encompassing 12,877 pregnancies were identified. Out of these, 16 (12%; 44 pregnancies) were excluded due to missing dates; thus, 116 (88%) with information on 12,833 pregnancies (99.7%) were analyzed. In the Additional file 1, sheet 1, references of the included and excluded articles are shown.

We sent e-mails asking for additional information about the time span of data collection to 12 authors; 9 (75.0%) [12–20] answered, and the studies were included in the charts; three did not answer, but one reported only the final date, and the study was included [21]. Four authors were contacted to clarify possible participant duplication; in two studies, there were no duplications [22, 23]; in one, partial overlap was confirmed [24], and the fourth could not confirm [25].

In Table 1, names of countries and number of studies are presented. In 61 studies (53%), individual data were provided; among these, 25 (41%) were reports of single cases; the remaining were case series ranging from two to 46 women. In the 55 case series studies that did not provide individual data, the smallest included nine women and the largest 8207. Excluded studies were slightly different from those included; there were no national registries nor case series with individual data.

**Table 1:** Number of studies by country and the absence/presence of individual data

Individual data		Country								Total
		Brazil	China	France	India	Italy	Spain	UK	USA	
No	*n*	2	24	3	0	4	5	3	14	55
	%	100	46	75	0	31	83	43	47	47
Yes	*n*	0	28	1	2	9	1	4	16	61
	%	0	54	25	100	69	17	57	53	53
Total		2	52	4	2	13	6	7	30	116

**Fig. 1:**
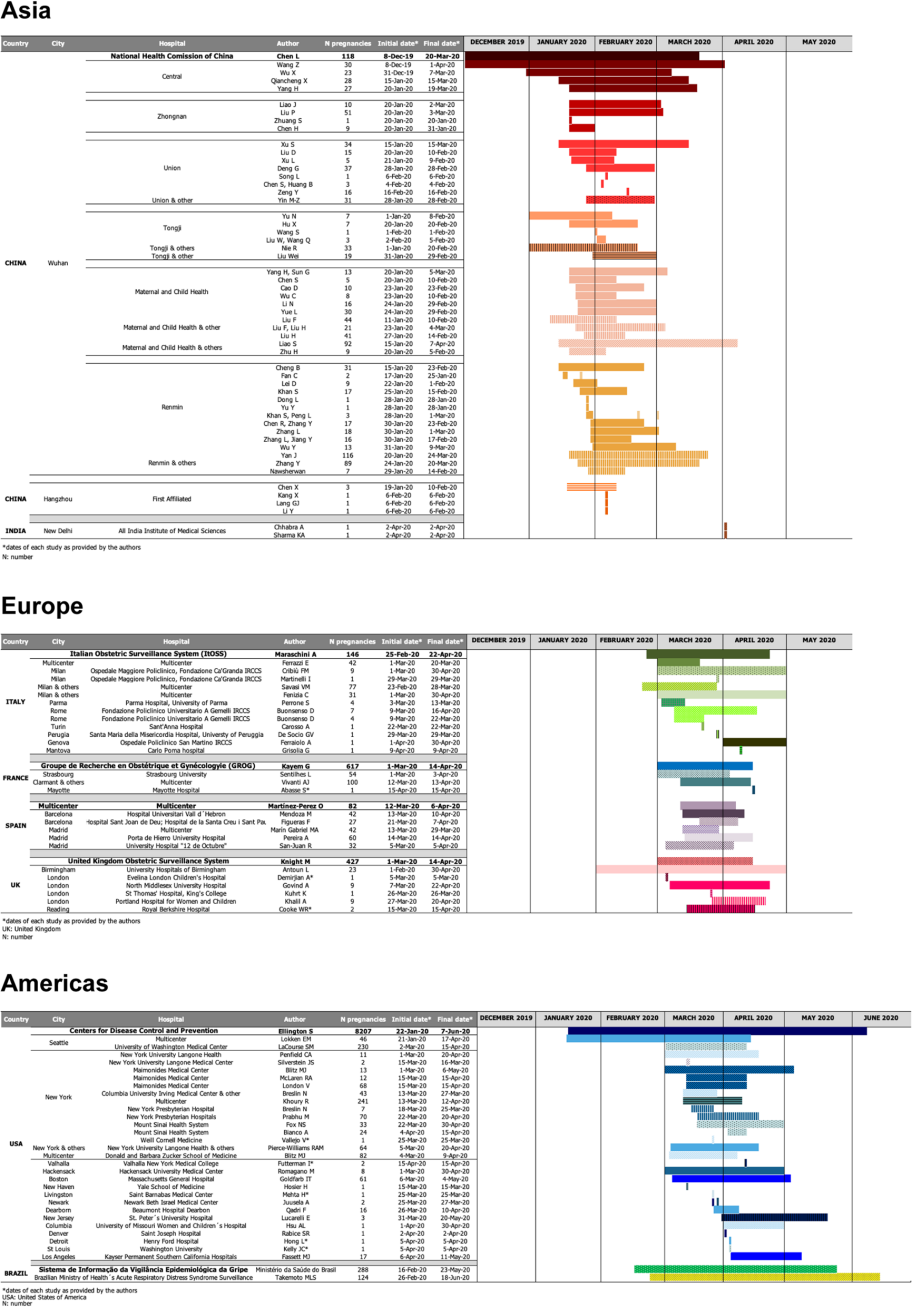
Gantt chart showing studies of COVID-19 in pregnant women to July 15, 2020

In Fig. 1, the Gantt chart with studies from all countries available by July 15, 2020, is shown. Scanning the chart, two issues came to mind. First, through the sequence of published studies, a timeline of the SARS-CoV-2 virus spread could be traced: initially in Asia, then Europe, and, finally, in the Americas. The second issue was a possible overlap of cases included in the studies. In Additional file 1, sheets 2 to 4, we present the raw data used to plot the Gantt chart.

Data from Wuhan’s hospitals are shown at the top of Fig. 1, as well as other studies from Asia. One may note that, across time, different reports could have included the same participants, because, for a particular hospital, some dates clearly overlapped. One large study [26] had a wide time frame that could include several smaller reports. The same pattern can be seen in series from Europe, plotted in the middle part of Fig. 1. Studies from Spain could encompass overlapping case series, since one of them was multicentric [23]. After contacting the authors, we confirmed that this multicentric study did not include the women of the two other studies [27, 28]. Regarding the Americas (bottom of the chart), several studies have dates within the time frame of the Centers for Disease Control and Prevention report [29]. If this large study is kept in the analysis, probably all smaller studies from the USA should be discarded. In two studies from Brazil [30, 31], initial and final dates of enrollment were almost the same, leading to the presumption that most cases might be the same; therefore, the official information of the Brazilian Health Ministry was elected [31].

When we evaluated, as an exercise, the rates of C-section in New York City, the incidence of the outcome changed (Additional file 2, sheets 1 and 2). Firstly, we estimated the crude C-section incidence including all studies; then, estimates of the C-section incidence were calculated by combining studies that clearly did not overlap. When more than one study was from the same hospital and within a similar time frame, incidence was recalculated by excluding these hospitals one by one, as done in a sensitivity analysis. Out of 14 studies, 11 could be included, presenting 208 C-sections among 532 women (incidence: 39%). Potential overlap may have occurred among cases of the Maimonides Medical Center (*n* = 3), those of the NY Presbyterian Hospital (*n* = 3), and those of the NY University Langone Health (*n* = 3). Consequently, five out of 11 studies (45%) were excluded due to presumed overlap of samples, representing 45/532 (8%) women. Notably, scrutinizing authorship was also significant; in Khoury’s et al. study [32], cases of Penfield et al. [33] and Pierce-Williams et al. [34] reports were explicitly included. We assumed that cases of Breslin et al. series [35, 36] were not included in Khoury’s because the latter authors did not reference Breslin’s studies. We calculated C-section incidence considering two models, each one excluding studies with potential duplication of cases (Additional file 2, sheet 2). The first one included four studies [12, 22, 32, 36], resulting in 141 C-sections among 355 women, incidence of 40%. In the second model, we included the studies with the largest series from each hospital [12, 22, 34, 36, 37], resulting in 87 C-sections among 246 women; incidence changed to 35%. Note that the 24 C-sections reported in Pierce-Williams’ et al. study [34] occurred in 12 hospitals, of which only three were in NY. Since we do not know the number of C-sections from each of the three hospitals, the last incidence estimate (35%) may be inaccurate.

## Discussion

The Gantt chart, detailing each study on COVID-19 in pregnant women, helped to identify possible double reporting of case(s) in different studies during the preparation of a systematic review. Plotting of the chart allowed investigators to visualize situations in which careful analysis is recommended to avoid double inclusion of the same participant in the results synthesis.

The Gantt chart can be used in several situations, each with a particular view, with the intention to evaluate “temporal dependencies” [38]. It was used to tell the “historiophoty” of a university department [39], and, more recently, to describe the clinical course of COVID-19 cases across time [40], and to describe cases of COVID-19 in some psychiatric facilities [41].

Duplicated reports are usually described and evaluated in meta-analysis of RCTs [5, 6]. Six patterns of duplication have been described, ranging from an article published in two different journals to what von Elm called a chaotic situation, in which “both study sample and outcomes of duplicates and main articles were different despite evidence that both articles originated from the same study. Definite confirmation was only possible through contact with the authors” [42].

Various techniques to identify duplicates have been proposed. One of them is an electronic identification of double titles, which is resolved with the use of a reference manager, such as EndNote, or the recently proposed “Systematic Review Assistant-Deduplication Module,” a program that yields better accuracy than EndNote for detecting duplicate titles [43]. Hints to detect duplication of studies when extracting data have been provided [44]. Visualizing possible duplications through the use of charts was recently brought forth; the authors showed graphical techniques, including Venn diagram [45]; thus, studying duplications is not out of date. Five aspects to “improve the reliability of meta-analysis” of any kind were suggested, and the first one indicates the following: “be vigilant about double counting” [6].

Overlap may be anticipated in case series reports when facing a new disease [46]. Clear reference to the FIRST (index) report in subsequent publications is mandatory. We found several examples: Yan et al. [47] included data of other four studies; Khoury et al. [32] reported that among their sample of 241 women, 84 (35%) were included in other publications [33, 34, 48]; and in two reports from Europe, the authors included four women in one study [49] and seven in other [24], but double reporting of four women was clearly stated in the second study and further clarified through contact with the author, who kindly provided individual data.

The influence of double reporting on the precision of estimates cannot be minimized. In the exercise shown here, the incidence of C-section in New York hospitals ranged from 35 to 40%; when we included all studies (*n* = 11), incidence was 39%; including the largest one and three others without evidence of overlap, the incidence was similar (40%); and when only the largest sample of each hospital was included (*n* = 5 studies), the incidence dropped to 35%; this difference might be clinically relevant.

Contacting the authors of studies may define the extent of the overlap; perhaps, one could go as far as asking for their individual level data. Our precision in the plotting of the Gantt chart improved, since most authors answered our requests upon dates and half of them, upon duplicated cases.

Plotting a Gantt chart has strengths: possible duplications can be easily visualized. For this, some information is crucial: the outcome of interest, the time span, and the specific setting of each study, such as clinic or hospital. Less relevant, but still necessary, is the ascertainment of studies authorship. It can also highlight potential duplication in situations in which partial results were published before the index paper, such as abstracts presented in congresses. Moreover, we could show the impact on the final figures of a more frequent outcome — the rates of C-section — after running a sensitivity analysis of some potential overlapping studies depicted in the chart.

Limitations must also be cited: the chart cannot be drawn when dates and/or settings are not provided. Here, we observed absent dates in some studies, which potentially could lead to bias; nevertheless, only 12% of the studies were excluded for this reason, representing loss of 44 pregnancies among the 12,877 eligible participants. Moreover, we observed a response rate of 75% from the authors we contacted regarding dates of data collection. Our interest was to demonstrate the existence (or not) of overlapping studies or inclusion of the same participants in more than one study; therefore, we did not consider the outcome of interest. Indeed, the Gantt chart helped us organize the multiple settings and time frames pictured in the myriad of studies published during the few first months of the pandemic. Finally, to our knowledge, the chart was not yet tested as a tool in detecting duplication in systematic reviews or meta-analysis of RCTs.

Eventually, our interest to better understand case duplications and how they could impact results actually converted us into true detectives, a feeling endorsed by other authors when they stated that researches may be doing a “detective work” while extracting data for a systematic review [44].

## Conclusions

The COVID-19 pandemic gave rise to a wave of (maybe) unintended double reporting, in an effort to understand the new disease. Double reporting can do more harm than good if it not expressly stated. The Gantt chart may help researchers in ascertaining possible overlap of individual cases, of case series, and, perhaps, even of randomized controlled trials.

## Supplementary Information

**Additional file 1.** Gantt Systematic Reviews.

**Additional file 2.** Number of C-section in pregnancies complicated by COVID-19, according to different authors and hospitals in New York.

## Data Availability

The datasets used for the analyses presented in this study are available as Additional file 1.
